# Utility of Comprehensive Genomic Profiling Tests for Patients with Incurable Pancreatic Cancer in Clinical Practice

**DOI:** 10.3390/cancers15030970

**Published:** 2023-02-03

**Authors:** Takuo Yamai, Kenji Ikezawa, Naotoshi Sugimoto, Makiko Urabe, Yugo Kai, Ryoji Takada, Tasuku Nakabori, Hiroyuki Uehara, Takahisa Kawamura, Kei Kunimasa, Sachiko Yamamoto, Toru Wakamatsu, Takuji Hayashi, Yoji Kukita, Fumie Fujisawa, Tazuko Inoue, Yuko Yamaguchi, Tomoyuki Yamasaki, Keiichiro Honma, Kazuyoshi Ohkawa

**Affiliations:** 1 Department of Hepatobiliary and Pancreatic Oncology, Osaka International Cancer Institute, Osaka 541-8567, Japan; 2 Department of Genetic Oncology, Osaka International Cancer Institute, Osaka 541-8567, Japan; 3 Department of Thoracic Oncology, Osaka International Cancer Institute, Osaka 541-8567, Japan; 4 Department of Gastrointestinal Oncology, Osaka International Cancer Institute, Osaka 541-8567, Japan; 5 Musculoskeletal Oncology Service, Osaka International Cancer Institute, Osaka 541-8567, Japan; 6 Department of Urology, Osaka International Cancer Institute, Osaka 541-8567, Japan; 7 Laboratory of Genomic Pathology, Osaka International Cancer Institute, Osaka 541-8567, Japan; 8 Department of Medical Oncology, Osaka International Cancer Institute, Osaka 541-8567, Japan; 9 Department of Endocrinology/Metabolism Internal Medicine, Clinical Examination, Osaka International Cancer Institute, Osaka 541-8567, Japan; 10 Department of Diagnostic Pathology and Cytology, Osaka International Cancer Institute, Osaka 541-8567, Japan

**Keywords:** comprehensive genomic profiling tests, pancreatic cancer, homologous recombination deficiency

## Abstract

**Simple Summary:**

Although comprehensive genomic profiling (CGP) tests have been covered under the Japanese national health insurance program, the utility and issues of CGP tests have not been clarified. We retrospectively reviewed 115 patients with incurable pancreatic cancer (IPC) who underwent CGP tests in a Japanese cancer referral center from November 2019 to August 2021. Eight cases (6.9%) were diagnosed as tumor mutation burden-high and/or microsatellite instability-high. The gene mutation rates of *KRAS/TP53/CDKN2A/SMAD4* were 93.0/83.0/53.0/25.2%, respectively. Twenty-five patients (21.7%) had homologous recombination deficiency (HRD)-related genetic mutations. Six patients (5.2%) underwent gene-matched therapy based on results of CGP tests. The median overall survival (OS) was significantly longer in the HRD (+) group. In multivariate analysis, HRD-related gene mutation was an independent prognostic factor associated with favorable OS. CGP tests for patients with IPC have the potential utility of detecting HRD-related gene mutations as prognostic factors as well as a therapeutic search.

**Abstract:**

Although comprehensive genomic profiling (CGP) tests have been covered under the Japanese national health insurance program since 2018, the utility and issues of CGP tests have not been clarified. We retrospectively reviewed 115 patients with incurable pancreatic cancer (IPC) who underwent CGP tests in a Japanese cancer referral center from November 2019 to August 2021. We evaluated the results of CGP tests, treatments based on CGP tests, and survival time. Eight cases (6.9%) were diagnosed as tumor mutation burden-high (TMB-H) and/or microsatellite instability-high (MSI-H). The gene mutation rates of *KRAS/TP53/CDKN2A/SMAD4* were 93.0/83.0/53.0/25.2%, respectively. Twenty-five patients (21.7%) had homologous recombination deficiency (HRD)-related genetic mutations. Four patients (3.5%) having TMB-H and/or MSI-H were treated with pembrolizumab, and only two patients (1.7%) participated in the clinical trials. Patient characteristics were not significantly different between patients with and without HRD-related gene mutations. The median OS was significantly longer in the HRD (+) group than in the HRD (−) group (749 days vs. 519 days, *p* = 0.047). In multivariate analysis, HRD-related gene mutation was an independent prognostic factor associated with favorable OS. CGP tests for patients with IPC have the potential utility of detecting HRD-related gene mutations as prognostic factors as well as a therapeutic search.

## 1. Background

Pancreatic cancer is a high-grade malignancy with a 5-year relative survival rate of less than 10% [1]. Surgical resection is the only curative treatment for pancreatic cancer. Early diagnosis of pancreatic cancer is extremely difficult [2,3], and approximately 80% of patients are diagnosed in the inoperable state due to local progression or distant metastasis at the time of presentation [4]. Recently, the use of pembrolizumab has been recommended for tumors with microsatellite instability-high (MSI-H) or a high tumor mutation burden (TMB-H). but is limited to a small number of cases [5,6]. Currently, chemotherapy remains the mainstay of treatment for advanced pancreatic cancer with the goal of prolonging prognosis. However, because its outcomes are not satisfactory, there is an urgent need to increase treatment options.

In recent years, a new treatment strategy for cancer has focused on cancer gene mutations. In the past, fluorescence in situ hybridization, copy number microarray, quantitative reverse transcription polymerase chain reaction, Sanger sequencing, fluorescence in situ hybridization and immunohistochemistry have been used in clinical practice to identify pathological genetic mutations [7]. Recently, with the advent of next-generation sequencers, it has become possible to comprehensively analyze large-scale genome information in a short time and at low cost, and multiple cancer-related genes are analyzed simultaneously in a single test as a new cancer companion diagnosis that can replace single-gene analysis [8]. Genomic stability, which leads to cancer gene mutations, is impaired in all cancers, including pancreatic ductal adenocarcinoma (PDAC), and can be broadly classified into chromosomal instability and microsatellite instability [9]. In pancreatic cancer, the nature of the cancer is most likely determined by chromosomal instability because the frequency of MSI-H is as low as 2.5% [10]. The results of chromosomal instability can be categorized into genetic mutations that are nearly universally found, such as *KRAS*, *TP53*, *CDKN2A* (*p16*) and *SMAD4* (*DPC4*), and low frequency mutations, such as *BRCA2* and *ERBB2* [11]. Mutations in genes related to homologous recombination repair, including *BRCA1/2*, *ATM* and *PALB2* genes, have been classified as low-frequency mutations. In pancreatic cancer, these low-frequency mutations result in a highly heterogeneous mutational background, giving each PDAC its unique features.

Homologous recombination deficiency (HRD) plays an important role in the cellular response to platinum-containing drugs [12,13]. The FOLFIRINOX (5-fluorouracil, leucovorin, oxaliplatin, and irinotecan) regimen has shown significantly better overall survival than gemcitabine as a first-line treatment in patients with metastatic pancreatic cancer [14]. Modified FOLFIRINOX could be a promising treatment option as a second-line therapy after gemcitabine plus nab-paclitaxel (GnP) failure [15]. On the other hand, in patients who underwent GnP and 5-fluorouracil/L-leucovorin plus nanoliposomal irinotecan, the efficacy of FOLFOX (5-fluorouracil, leucovorin, oxaliplatin) as salvage chemotherapy has been reported to be limited to patients with HRD-associated gene mutations [16]. The evaluation of mutations in HRD-associated genes may be useful in making treatment decisions. With the improvement of genetic analysis capabilities using NGS, comprehensive genomic profiling (CGP) tests are now being implemented in clinical practice. Comprehensive analysis of HRD-related genes, as well as many other genes and microsatellite instability, is now available.

In Japan, insurance coverage of cancer genome medicine was applied in June 2019 [17]. Two types of CGP tests (OncoGuide NCC Oncopanel System and FoundationOne CDx Cancer Genome Profile) using tissue samples are now widely available under the national health insurance system [17,18]. However, for patients with advanced pancreatic cancer whose survival time is expected to be limited, some problems remain to be solved, such as drug accessibility. The usefulness of the CGP test in clinical practice has not been fully elucidated. In this study, we analyzed the results and clinical outcomes of the CGP test in patients with incurable pancreatic cancer (IPC) in our institution and evaluated the utility of the CGP test in clinical practice.

## 2. Material and Methods

### 2.1. Study Design, Patients and Collecting Data

**Figure 1:**
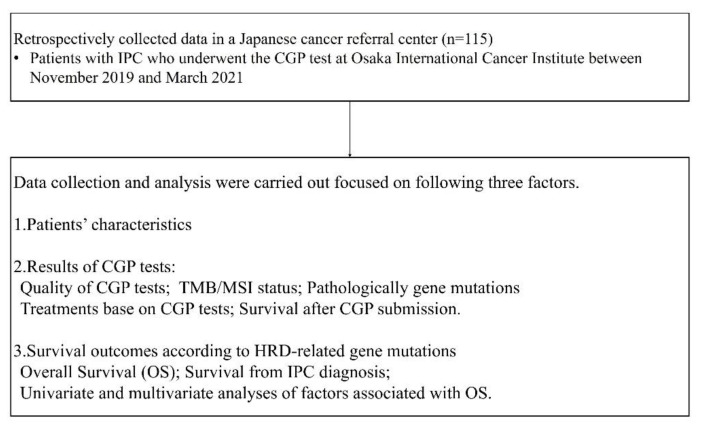
Study design. IPC, incurable pancreatic cancer; CGP, comprehensive genomic profiling; TMB, tumor mutation burden; MSI, microsatellite instability; HRD, homologous recombination deficiency.

The study design is shown in Figure 1. This study was a retrospective study of 115 IPC patients who underwent the CGP test in a Japanese cancer referral center (Osaka International Cancer Institute) between November 2019 and March 2021. All patients were histologically diagnosed as adenocarcinoma. For each patient, clinical data were extracted from medical records. Follow-up data from patients were censored on 28 February 2022. Data collection and analysis were carried out focused on the following three factors.

1. Patients’ characteristics

The following clinical parameters were obtained: age, sex, Eastern Cooperative Oncology Group (ECOG), performance status (PS), resectability at the initiation of treatment for pancreatic cancer, laboratory data (including white blood cells (WBC), hemoglobin (Hb), platelet (Plt), albumin and carbohydrate antigen 19-9 (CA19-9)), treatment regimens and treatment efficacy. Resectability at the initiation of treatment for pancreatic cancer was classified as resectable (R), borderline resectable (BR), locally advanced unresectable (UR-LA) and metastatic unresectable (UR-M) according to National Comprehensive Cancer Network guidelines [19]. For the patients initially diagnosed with R or BR PC, CGP was performed after diagnosis as incurable or recurrence after surgery.

2. Results of CGP tests

In this study, the CGP tests included the OncoGuide™ NCC Oncopanel System (Sysmex Corporation, Hyogo, Japan) and FoundationOne CDx (Foundation Medicine, Cambridge, MA, USA), both of which were approved by national insurance in Japan. The type of CGP test was decided based on the clinical judgment of each attending physician and patients’ will. All CGP tests were performed with formalin-fixed paraffin-embedded specimens. The specimens used for CGP tests were those deemed appropriate by each attending physician and pathologists at our hospital. The method of tissue sample collection was determined by the attending physicians to be appropriate. All cases were reviewed by expert panels, which were molecular tumor board meetings consisting of experts in various fields, including oncologists, geneticists, pathologists, bioinformaticians and genetic counselors [20]. Genetic variants based on the report were again judged by the expert panel for their pathological significance. FoundationOne CDx, which accounts for 80.9% of cases in this study, is performed on only tumor tissue samples. The tumor-derived gene mutations include both somatic and germline mutations. Due to the nature of the panel tests, it is difficult distinguish whether the detected genes were somatic or germline mutations. Therefore, in this study, genes determined to be pathological changes were defined as pathologically mutated genes of tumors. In addition, HRD-related genes were defined as pathogenic alterations of the following 17 homologous recombination genes as previously reported: *ATM*, *BAP1*, *BARD1*, *BLM*, *BRCA1*, *BRCA2*, *BRIP1*, *CHEK2*, *FAM175A*, *FANCA*, *FANCC*, *NBN*, *PALB2*, *RAD50*, *RAD51*, *RAD51C* and *RTEL1* [21]. Based on the report approved by the expert panel as the results of CGP tests, we extracted quality of tests, TMB/MSI-status, the number of pathological gene mutations, the mutation rates of KRAS/TP53/CDKN2A/SMAD4/HRD-related genes and treatments based on CGP tests. In addition, we evaluated survival after CGP submission. Survival after submission of CGP tests was defined as the period from the submission of CGP tests to the date of death.

3. Survival outcomes according to HRD related gene mutations

The following two survival outcomes were evaluated: overall survival (OS) and survival from IPC diagnosis. OS was calculated from the start date of 1st-line (for UR-LA or UR-M PC), the date of upfront surgery (for one case with R PC) or the start date of neoadjuvant (for the other cases with R or BR PC) chemotherapy to the date of death. Survival after diagnosis of IPC was calculated from the start date of diagnosis as incurable or recurrence after surgery to the date of death. We also examined the prognostic factors associated with OS.

This study was conducted in accordance with the Declaration of Helsinki. Ethical approval was obtained from the Ethical Review Committee of the Osaka International Cancer Institute (20148-3). The requirement for informed consent was waived by the opt-out method of our hospital’s website.

### 2.2. Statistical Analysis

Categorical variables are described as percentages, and continuous variables are presented as the median and range. Patient characteristics and treatment outcomes were compared between HRD-related mutation-positive patients, the HRD (+) group and the other patients (HRD (−) group) using Fisher’s exact tests and the χ^2^ test with Yate’s correction for categorical variables and the Mann-Whitney U test for continuous variables. OS, survival after diagnosis of IPC and survival after submission of CGP tests were compared using the Kaplan-Meier curve and log-rank test. Univariate and multivariate analyses were analyzed by using the Cox proportional hazard model. The hazard ratio (HR) and 95% confidence interval (CI) were calculated. Factors with *p* values less than 0.05 in univariate analysis were included in multivariate Cox models. For the *p* value, the significance level was defined as 0.05. Statistical analyses were performed using JMP Ver. 14.0 (SAS Institute, Cary, NC, USA).

## 3. Results

### 3.1. Characteristics of CGP Tests

Patient characteristics are summarized in Table 1. The median age (range) was 63 years (37–80 years), and 63 patients (54.8%) were male. Sixty-five patients (56.5%) were diagnosed with UR-M at the initiation of treatment for pancreatic cancer. Panel tests were submitted during first-line treatment in 30 patients (26.1%), after disease progression of first-line treatment in 59 patients (51.3%), and after disease progression of second-line treatment or later in 26 patients (22.6%). Primary organs of samples submitted for CGP tests were the pancreas in 51 patients (44.4%) and liver in 45 patients (39.1%). Specimens obtained by endoscopic ultrasound-guided fine-needle aspiration (EUS-FNA) or percutaneous needle biopsy were used in 96 patients (83.5%), while surgical specimens were used in 24 patients (20.9%). FoundationOne CDx and NCC Oncopanel were used in 93 patients (80.9%) and 22 patients (19.1%), respectively.

### 3.2. Results of CGP Tests

The results of the CGP test are summarized in Table 2. The tissue samples of 98 patients (85.2%) satisfied the criteria for FoundationOne CDx or NCC Oncopanel, while samples of the other 17 patients (14.8%) were evaluated as “qualified”, which meant the reduction of sensitivity of TMB, MSI, gene mutations or copy-number alterations. Eight patients (6.9%) were diagnosed with TMB-H and/or MSI-H. The median number of pathological gene mutations examined in the expert panel was 4 (range, 1–10). The gene mutation rates of *KRAS*, *TP53*, *CDKN2A* and *SMAD4* were 107 patients (93.0%), 96 patients (83.0%), 61 patients (53.0%) and 29 patients (45.2%), respectively. In addition, 25 patients (21.7%) had HRD-related genetic mutations. These included *BRCA1/2* (11 patients (9.6%)), *ATM* (4 patients (3.5%)), *RAD51C* (3 patients (2.6%)), *PALB2* (2 patients (1.7%)), and other mutations (5 patients (4.3%)). In this study, a total of six patients (5.2%) underwent gene-matched therapy based on results of CGP tests. Four patients having TMB-H and/or MSI-H (3.5%) were treated with pembrolizumab. The other two patients (1.7%) participated in the clinical trials regarding gene-matched therapy: one with a *KRASG12C* inhibitor and the other with an *ROS1* inhibitor. From our cohort, the median survival after submission of the CGP test was 182 days [95% CI: 150–227 days]. A total of 16 patients (13.9%) died within 90 days (Supplementary Figure S1).

### 3.3. Survival Outcomes According to HRD-Related Gene Mutations

Next, we examined the clinical significance of HRD-related gene mutations. The patients’ characteristics according to HRD-related gene mutations are summarized in Table 3. The median age was similar in both groups (HRD (+), 61 years (range: 38–78 years) vs. HRD (−), 65 years (range: 37–80 years), *p* = 0.585). There were no significant differences in sex, resectability at the initiation of treatment for pancreatic cancer, the frequency of TMB-H or MSI-H, PS, the rate of curative resection and the levels of WBC, Hb, Plt, albumin and CA19-9 between the two groups. Although there was no significant difference between the two groups, the frequency of patients who underwent platinum-containing regimens was higher in the HRD (+) group (84.0%) than in the HRD (−) group (66.7%) (*p* = 0.136). In summary, there were no significant differences in patient background or treatment between the HRD (+) group and HRD (−) group.

### 3.4. Treatment Outcomes According to HRD-Related Genetic Mutations

**Figure 2:**
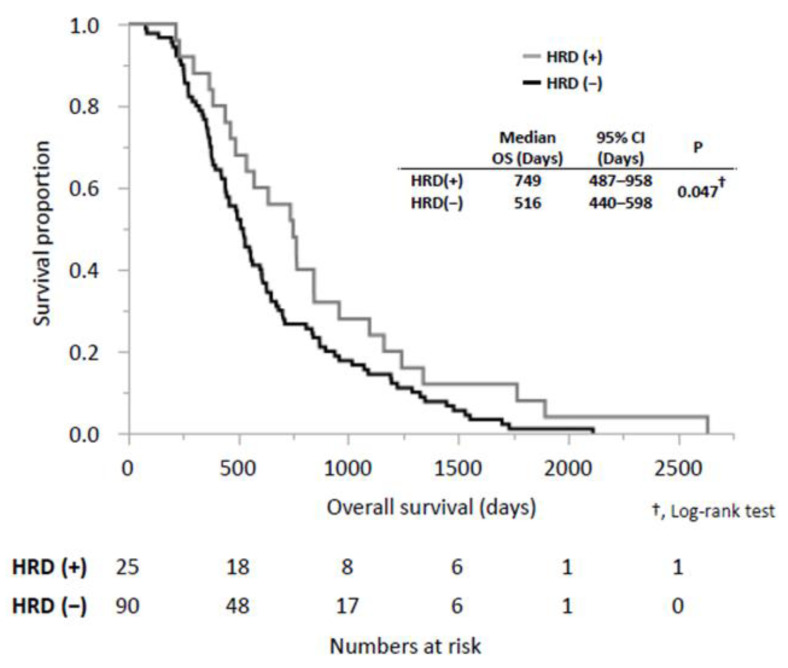
Comparison of overall survival (OS) between the patients who had homologous recombination deficiency (HRD)-related genetic mutations (HRD (+)) and those who did not (HRD (−)). †, Log-rank test; OS, overall survival; HRD, homologous recombination deficiency; CI, confidence interval.

The median OS and survival from IPC diagnosis were significantly longer in the HRD (+) group than in the HRD (−) group (OS: 749 days [95% CI: 487–958 days] vs. 516 days (95% CI: 440–598 days], *p* = 0.047; survival from IPC diagnosis: 636 days [95% CI: 422–958 days] vs. 441 days (95% CI: 382–510 days], *p* = 0.002) (Figure 2 and Figure S2). Finally, we examined the prognostic factors associated with OS (Table 4). In a univariate analysis of OS, three variables were significantly associated with OS: resectability at the initiation of treatment for pancreatic cancer (UR-M vs. R, BR, and UR-LA) (HR, 1.82; 95% CI, 1.25–2.66; *p* = 0.002), HRD-related gene mutation (HR, 0.63; 95% CI, 0.44–0.99; *p* = 0.049) and baseline Hb levels (HR, 1.58; 95% CI, 1.08–2.30; *p* = 0.018). Multivariate analysis was performed using these variables. HRD-related gene mutation was identified as a statistically significant independent predictor of OS (HR, 0.60; 95% CI, 0.34–0.96; *p* = 0.035), as with resectability at the time of diagnosis (HR 1.97, 95% CI, 1.34–2.92, *p* < 0.001) and Hb levels (HR 1.54, 95% CI 1.06–2.23, *p* = 0.025).

## 4. Discussion

In this study, we evaluated the utility of CGP tests for IPC patients in clinical practice, which mostly reflected pathogenic mutations in each pancreatic cancer because the quality criteria for CGP were satisfied in approximately 85% of the patients. Furthermore, mutation rates of the “big 4” genes (*KRAS*, *TP53*, *CDKN2A* and *SMAD4*) were detected at the same proportions as previously reported [6]. These results showed that CGP tests in this study adequately detected pathological gene mutations. Based on the results described above, we discuss the utility of CGP tests in clinical practice, focusing on the following two points.

First, the number of patients who underwent gene-matched therapies based on CGP tests was limited. While four out of eight patients who were diagnosed as TMB-H and/or MSI-H underwent pembrolizumab, only two patients (1.7%) participated in the clinical trials. Tumor MSI status and TMB have been shown to play a significant role in the therapeutic efficacy of immune checkpoint inhibitors [22,23]. The Food and Drug Administration approved pembrolizumab, an antibody against the programmed cell death-1 (PD-1) protein, for the treatment of patients with MSI-H solid tumors in 2017 and TMB-H solid tumors in 2020 [24,25]. In Japan, the use of pembrolizumab was approved for patients with MSI-H solid tumors in December 2018 and for those with TMB-H solid tumors in February 2022 [25,26]. The percentages of TMB-H and MSI-H in pancreatic cancer have been reported to be 1.4–27.9% and 0–1.3%, respectively [18]. Although the frequencies of TMB-H and/or MSI-H were similar to those in previous reports, half of the patients with TMB-H and/or MSI-H were not treated with pembrolizumab in the present study. This may be due to patient factors such as poor general condition and complications. In this study, a total of six patients (5.2%) underwent gene-matched therapy based on results of CGP tests. While four patients (3.5%) having TMB-H and/or MSI-H were treated with pembrolizumab, only two patients (1.7%) participated in clinical trials. The rate of patients who applied to gene-matched therapy based on CGP tests has thus far been reported to be 5–10% [21,27]. In our study, the rate of gene-matched therapy was similar to that of previous reports. A recent report from the United States revealed that patients with IPC who underwent gene-matched therapy based on molecular profiling showed a significantly favorable prognosis compared with those who did not [21]. Hence, it is important to search for pathological mutations and gene-matched therapies to prolong the survival time of patients with IPC. However, the opportunity to participate in clinical trials seems to be limited in Japan. One reason for this may be due to the small number of investigational drugs and clinical trials, as well as regional differences in the sites where clinical trials are conducted. Another reason may be the worsening of the patient’s general condition due to disease progression. In Japan, CGP tests are covered by health insurance in patients with solid tumors who have completed standard therapy and those who are expected to complete it [18,28]. Indeed, CGP was submitted after disease progression of first-line treatment or later in our 85 patients (73.9%). However, late timing of CGP test submission may decrease the opportunity to undergo gene-matched therapy because 16 patients (13.9%) died within 90 days after submission of CGP tests in this study. Although second-line chemotherapy offered a survival benefit in patients with advanced pancreatic cancer, it was still limited; OS and PFS have been shown to be 4.1–9.9 and 1.4–3.1 months [29,30,31,32]. Although little evidence has been established regarding the significance of gene testing from an early stage in solid tumors [28], it is preferable to submit CGP at an earlier time, especially in patients with IPC, because of rapid disease progression. Suitable timing of CGP tests may lead to decisions regarding treatment under favorable patient conditions.

Second, this study showed the frequency of HRD-related gene mutations and their significance as prognostic factors associated with OS in patients with IPC. The rate of overall HRD-related gene mutation was 21.7%, similar to the rate of a previous report regarding HRD-related genes in patients with advanced pancreatic cancer (19%), which included 15% germline mutations and 4% somatic mutations [21]. In the present study, IPC patients with HRD-related gene mutations had significantly longer overall survival than those without the mutations. Furthermore, in multivariate analysis, HRD-related gene mutation was identified as an independent prognostic factor for OS. The results of previous nonrandomized clinical trials infer HRD-related gene mutation as a predictor of therapeutic response for platinum-based chemotherapy in patients with advanced pancreatic cancer [21,33]. The results of our study suggested two possible benefits of checking HRD status: (1) if a patient underwent platinum-based regimens such as FOLFIRINOX, the management of adverse effects associated with platinum-containing drugs including peripheral neuropathy is important for their long-term use; (2) if a non-platinum regimens are used as the first-line regimen, the use of platinum-based regimens are preferential as a second-line regimen because the patients with HRD-related gene mutation may miss the opportunity to use platinum-containing drugs at later lines, primarily due to disease progression. HRD status could be an important indicator in selecting a second-line treatment regimen. Taken together, the results of HRD gene status obtained by the CGP test in clinical practice can be used as a predictor of efficacy for platinum-based chemotherapy, suggesting a further advantage of earlier submission of CGP tests, which can contribute to the appropriate selection of chemotherapeutic regimens. Obtaining CGP results before starting second-line chemotherapy could contribute to decision-making regarding chemotherapeutic regimens.

This study had several limitations. First, this study was a retrospective study performed at our single hospital; hence, further multicenter studies with a larger number of patients will be needed. Second, clinical outcomes were analyzed only in the patients who underwent CGP tests. Thus, patients with IPC that could not undergo CGP tests were not included in this study. Third, our result showed that the number of patients receiving gene-matched therapies was limited. One of reasons for this was accessibility to clinical trials. Some trials were conducted only in remote areas from our hospital, even if the CGP test showed targeted gene mutations. The number of patients participating in a clinical trial may be changed in different areas.

## 5. Conclusions

In conclusion, CGP tests for patients with IPC in clinical practice have limited utility of searching for gene-matched therapies covered by insurance and clinical trials; however, more clinical trials in the future will be expected to increase the chance of gene-matched treatments based on CGP tests. Moreover, our report reveals that CGP tests for patients with IPC have the potential utility of detecting HRD-related genetic mutations as a prognostic factor. However, this study was a single-center, retrospective analysis; hence, further multicenter studies with a larger number of patients will be needed.

## Figures and Tables

**Table 1:** Patient characteristics of patients with incurable pancreatic cancer who underwent comprehensive genomic profiling (CGP) tests.

Number of patients, *n*	115
Median age (range), y.o	63 (37–80)
Sex	
Male, *n* (%)	63 (54.8)
Female, *n* (%)	52 (45.2)
Diagnosis at the start of treatment	
R or BR, *n* (%)	26 (22.6)
UR-LA, *n* (%)	24 (20.9)
UR-M, *n* (%)	65 (56.5)
Timing of submitting cancer gene panel tests	
During 1st-line treatment, *n* (%)	30 (26.1)
After disease progression of 1st-line treatment, *n* (%)	59 (51.3)
After disease progression of 2nd-line treatment, *n* (%)	26 (22.6)
Samples for CGP tests	
Pancreas, *n* (%)	51 (44.4)
Liver, *n* (%)	45 (39.1)
Lymph node, *n* (%)	7 (6.1)
Gastrointestinal tract, *n* (%)	5 (4.3)
Lung, *n* (%)	4 (3.5)
Peritoneum, *n* (%)	3 (2.6)
Sampling methods	
EUS-FNA, *n* (%)	44 (38.3)
Percutaneous needle biopsy, *n* (%)	42 (36.5)
Surgery, *n* (%)	24 (20.9)
Forceps biopsy, *n* (%)	5 (4.3)
Kinds of cancer gene panel tests	
FoundationOne CDx, *n* (%)	93 (80.9)
NCC Oncopanel, *n* (%)	22 (19.1)

R, resectable; BR, borderline resectable; UR-LA, unresectable-locally advanced; UR-M, unresectable-metastatic; EUS-FNA, endoscopic ultrasound-guided fine-needle aspiration.

**Table 2:** The results of comprehensive genomic profiling (CGP) tests.

Quality control	
Pass/met the criteria, *n* (%)	98 (85.2)
Qualified, *n* (%)	17 (14.8)
TMB-H or MSI-H, *n* (%)	8 (6.9)
Pathological gene mutations (range), *n*	4 (1–10)
KRAS mutations, *n* (%)	107 (93.0)
TP53 mutations, *n* (%)	96 (83.0)
CDKN2A mutations, *n* (%)	61 (53.0)
SMAD4 mutations, *n* (%)	29 (45.2)
HRD-related genes mutations, *n* (%)	25 (21.7)
BRCA1/2 mutations, *n* (%)	11 (9.6)
ATM mutations, *n* (%)	4 (3.5)
RAD51C mutations, *n* (%)	3 (2.6)
FANCA mutations, *n* (%)	2 (1.7)
Others mutations, *n* (%)	5 (4.3)
Administration of pembrolizumab, *n* (%)	4 (3.5)
Clinical Trial Participation, *n* (%)	2 (1.7)

TMB-H, tumor mutation burden-high; MSI-H, microsatellite instability-high; HRD, homologous recombination deficiency.

**Table 3:** Comparison of patient characteristics between the patients who had homologous recombination deficiency (HRD)-related genetic mutations (HRD (+)) and those who did not (HRD (−)).

	HRD (+)	HRD (−)	*p*-Value
Number of patients, *n*	25	90	
Median age (range), y.o.	61 (38–78)	64 (37–80)	0.585 ^†^
Sex			0.822 ^§^
Male, *n* (%)	13 (52.0)	50 (55.6)	
Female, *n* (%)	12 (48.0)	40 (44.4)	
Operability at the time of diagnosis			
R or BR, *n* (%)	5 (20.0)	22 (24.4)	0.658 ^§^
UR-LA, *n* (%)	6 (42.0)	18 (20.0)	
UR-M, *n* (%)	14 (56.0)	50 (55.6)	
TMB-H or MSI-H, *n* (%)	1 (4.0)	7 (7.8)	1.000 ^§^
Performance status			0.691 ^§^
0	16 (64.0)	60 (66.7)	
1-	5 (20.0)	21 (23.3)	
NA	4 (16.0)	9 (10.0)	
Platinum-based regimen, *n* (%)	21 (84.0)	60 (66.7)	0.136 ^§^
Curative resection, *n* (%)	4 (16.0)	24 (26.7)	0.429 ^§^
Median WBC (range), /μL	5380 (2970–10,490)	5420 (2530–19,910)	0.929 ^†^
Median Hb (range), g/dL	12.8 (9.4–15.0)	12.5 (8.2–15.1)	0.265 ^†^
Median Platelet (range), 10^4^/μL	20.5 (15.1–44.9)	22.8 (9.7–49.7)	0.610 ^†^
Median Albumin (range), mg/dL	3.8 (2.8–4.5)	3.8 (2.3–5.5)	0.187 ^†^
Median CA19-9 (range), mg/dL	503 (2–100,000)	368 (2–100,000)	0.741 ^†^

†, Wilcoxon test; §, Fisher’s test. HRD, homologous recombination deficiency; R, resectable; BR, borderline resectable; UR-LA, unresectable-locally advanced; UR-M, unresectable-metastatic; TMB-H, tumor mutation burden-high; MSI-H, microsatellite instability-high; NA, not assessed; WBC, white blood cell; Hb, hemoglobin; CA19-9, carbohydrate antigen 19-9.

**Table 4:** Univariate and multivariate analyses of factors associated with overall survival (OS).

	Univariate Analysis			Multivariate Analysis		
	HR	95% CI	*p*-Value	HR	95% CI	*p*-Value
Age (>70 vs. ≤70, y.o.)	0.65	0.43–1.00	0.051			
Male vs. Female	0.73	0.51–1.08	0.114			
PS (1- and NA vs. 0)	1.43	0.96–2.11	0.078			
Operability at the time of diagnosis. (UR-M vs. R, BR, and UR-LA)	1.82	1.25–2.66	**0.002**	1.97	1.34–2.92	**<0.001**
HRD related gene mutation (HRD (+) vs. HRD (−))	0.63	0.40–0.99	**0.049**	0.60	0.34–0.96	**0.035**
Platinum-based regimen (Y vs. N)	0.97	0.64–1.50	0.876			
Baseline WBC (>5420 vs. ≤5420, /μL)	0.92	0.63–1.34	0.673			
Baseline Hb (<12.7 vs. ≥12.7, g/dL)	1.58	1.08–2.30	**0.018**	1.54	1.06–2.23	**0.025**
Baseline Platelet (<22.6 vs. ≥22.6, ×10^4^/μL)	0.98	0.67–1.43	0.915			
Baseline albumin (<3.8 vs. ≥3.8, U/mL)	1.45	0.99–2.12	0.056			
Baseline CA19-9 (>400 vs. <400, U/mL)	1.41	0.96–2.06	0.078			

OS, overall survival. PS, performance status; NA, not assessed; R, resectable; BR, borderline resectable; UR-LA, unresectable-locally advanced; UR-M, unresectable-metastatic; WBC, white blood cell; Hb, hemoglobin; CA19-9, carbohydrate antigen 19-9.

## Data Availability

The data underlying the findings of our study cannot be publicly shared because of the nature of ethical approvals for the study. The data are available for researchers who meet the criteria of the Osaka International Cancer Institute Ethics Committee (via email to takuo.yamai@oici.jp) for access to confidential data.

## Supplementary Materials

The following supporting information can be downloaded at: https://www.mdpi.com/article/10.3390/cancers15030970/s1. Figure S1: Kaplan-Meier survival curves for survival after submission of comprehensive genomic profiling (CGP) tests. CI, confidence interval; Figure S2: Comparison of survival from IPC diagnosis between the patients who had homologous recombination deficiency (HRD)-related genetic mutations (HRD (+)) and those who did not (HRD (−)). IPC, incurable pancreatic cancer; OS, overall survival; HRD, homologous recombination deficiency; CI, confidence interval..
