# Supplementary material for: Utility of Comprehensive Genomic Profiling Tests for Patients with Incurable Pancreatic Cancer in Clinical Practice
Source: Cancers (Basel). 2023 Feb 3;15(3):970. doi: 10.3390/cancers15030970 (PMC9913675; doi:10.3390/cancers15030970)
Supplement: Supplementary file 1 [file cancers-15-00970-s001.zip › cancers-2154167-supplementary.pdf]

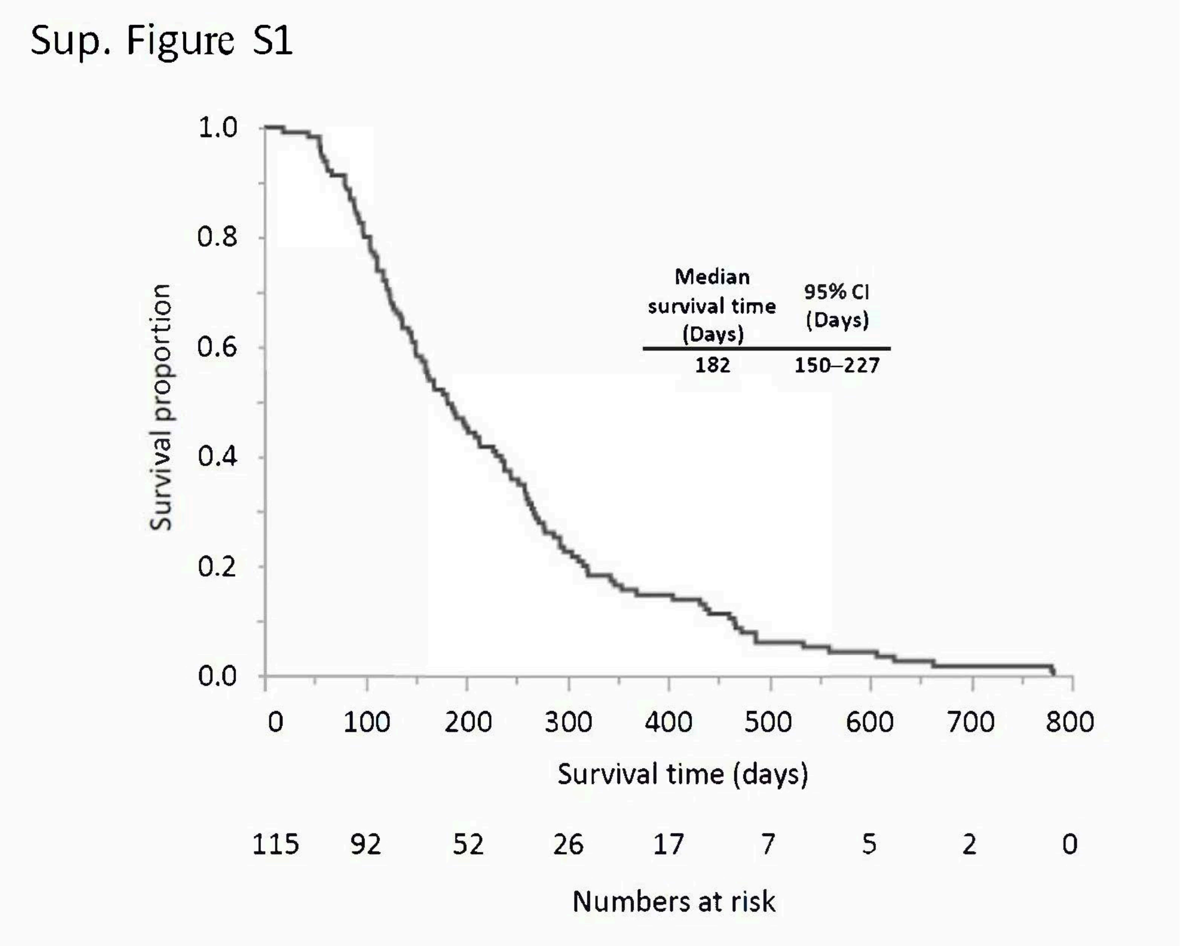

Supplementary Figure S1

Kaplan–Meier survival curves for survival after submission of comprehensive genomic profiling (CGP) tests. CI, confidence interval.

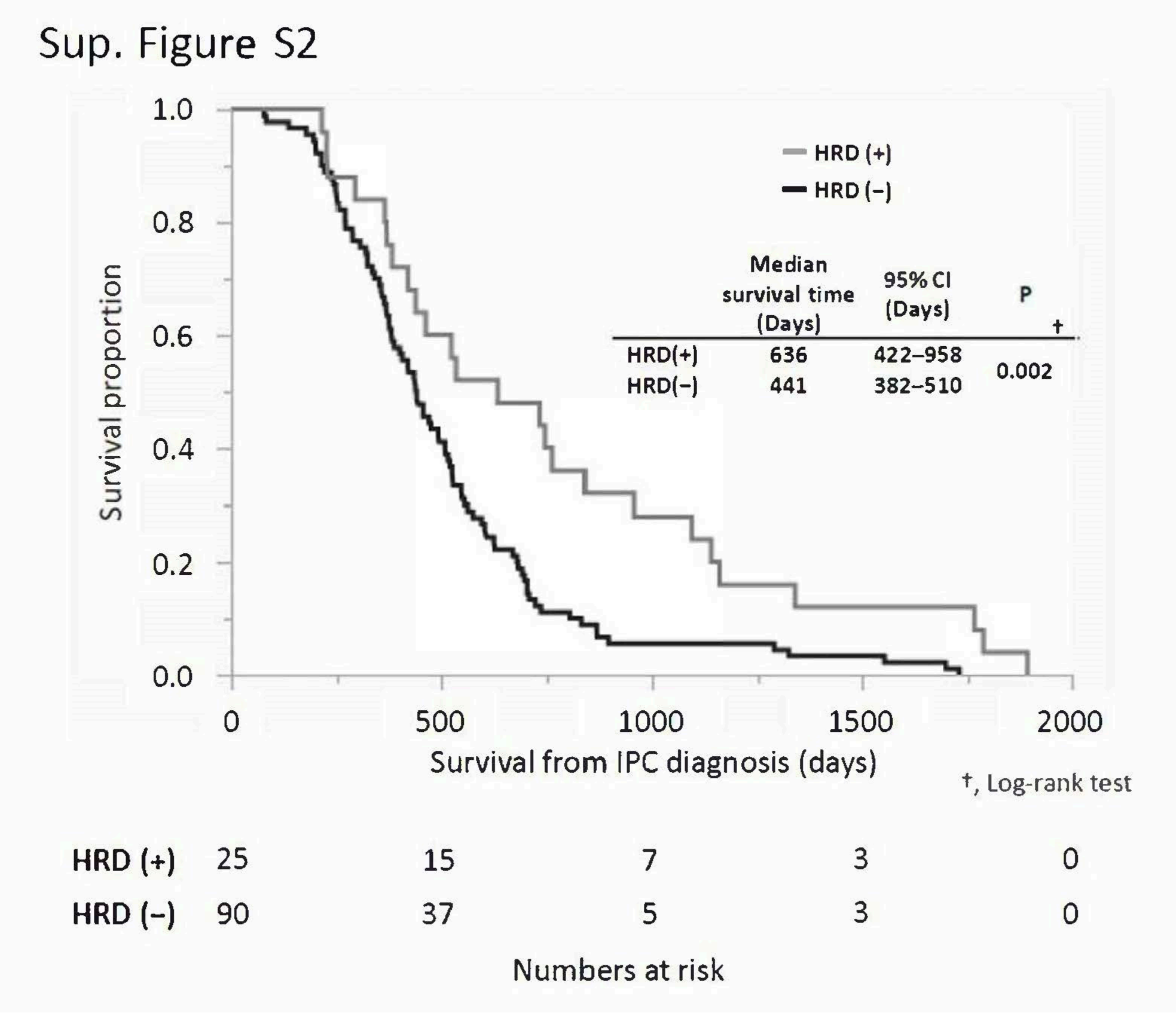

Supplementary Figure S2

Comparison of survival from IPC diagnosis between the patients who had homologous recombination deficiency (HRD)-related genetic mutations (HRD (+)) and those who did not (HRD (－)).

IPC, incurable pancreatic cancer; OS, overall survival; HRD, homologous recombination deficiency; CI, confidence interval.
